# Supplementary material for: Competing Endogenous RNA of Snail and Zeb1 UTR in Therapeutic Resistance of Colorectal Cancer
Source: Int J Mol Sci. 2021 Sep 3;22(17):9589. doi: 10.3390/ijms22179589 (PMC8431469; doi:10.3390/ijms22179589)
Supplement: Supplementary file 1 [file ijms-22-09589-s001.zip › ijms-1319213-supplementary.pdf]

Supplementary Materials

a

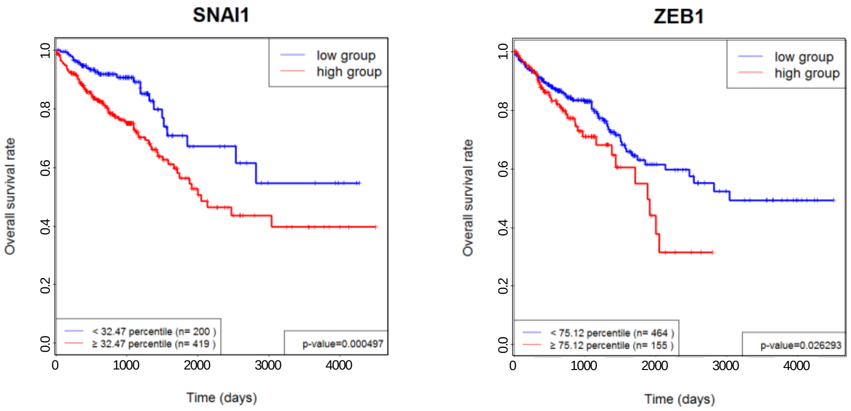

b

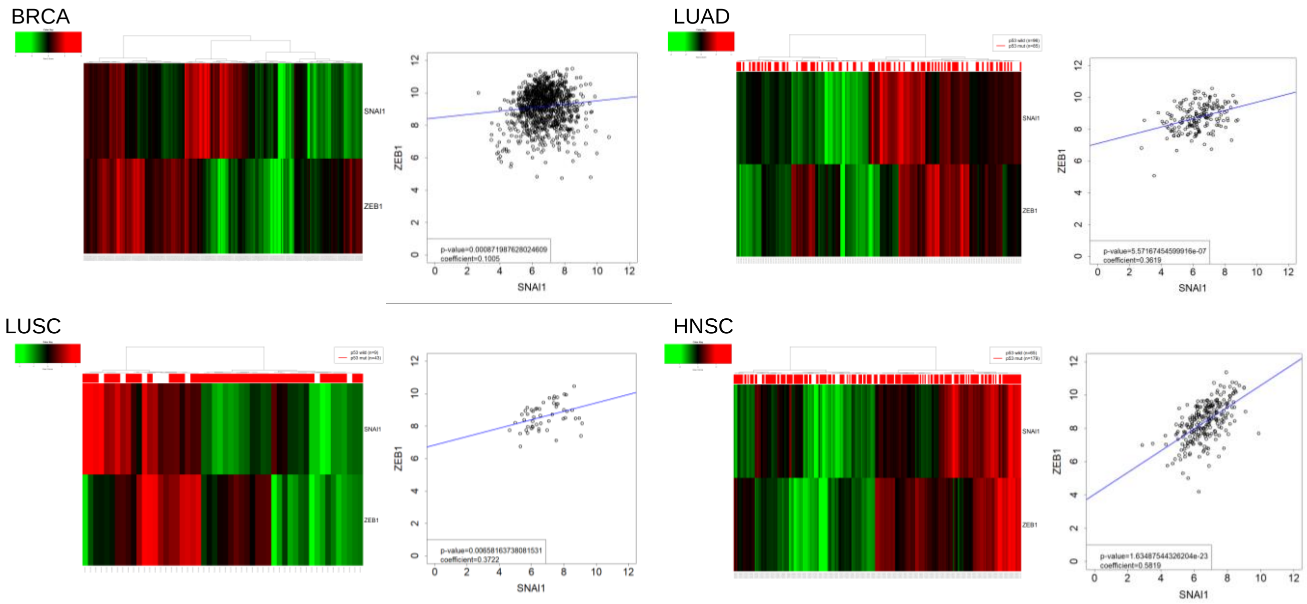

**Cancer types**	**Dataset (n)**	**Correlation coefficient (*p* value)**
Breast cancer (BRCA)	mRNA (1093)	0.1005 (*p*=0.0008)
Lung adenocarcinoma (LUAD)	n(mRNA ∩ miRNA ∩mutation) = 181	0.3619 (*p*=5.7e-07)
Lung squamous cell carcinoma (LUSC)	n(mRNA ∩ miRNA ∩ mutation) = 52	0.3722 (*p*=0.0066)
Head & neck squamous cell carcinoma (HNSC)	n(mRNA ∩ miRNA ∩ mutation) = 244	0.5819 (*p*=1.63e-23)

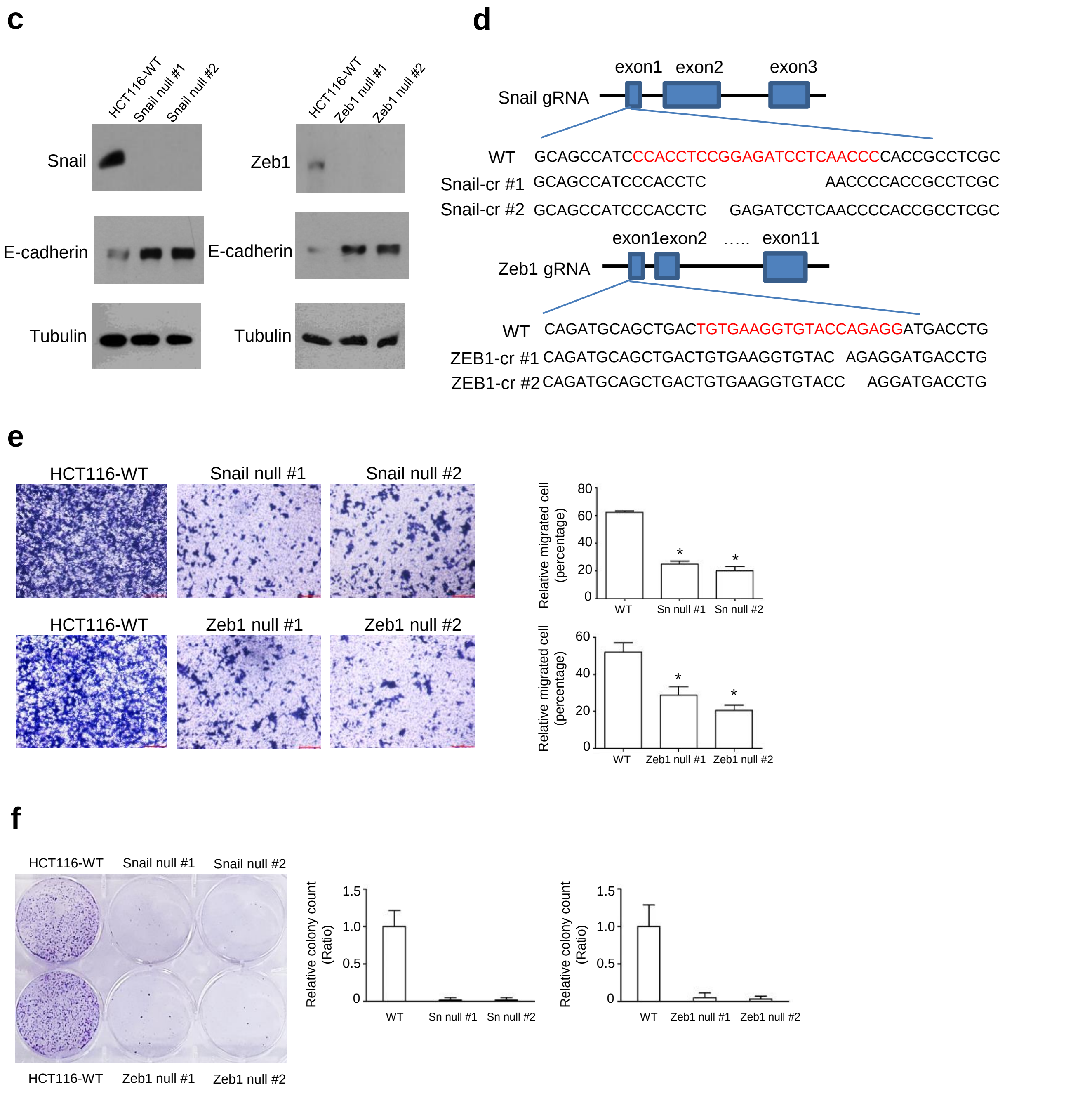

Figure S1. Reciprocal interaction between Snail and Zeb1:

(**a**) Survival rate of patients with different expression of Snail and Zeb1; (**b**) An supervised hierarchical clustering of genes associated with Snail and Zeb1 expression profiles in other type of cancers. In the heatmap, red denotes higher relative expression, whereas green indicates lower relative expression, with degree of color saturation reflecting the magnitude of the log expression signal; (**c**) Immunoblot analysis of Snail (Left panel), Zeb1 (Right panel) and E-cadherin (Both panel) in whole lysate, after respective knockout of Snail and Zeb1 in HCT116. Representative blots are shown from at least two independent experiments; (**d**) Schematic illustration of Snail1 and Zeb1 gene structure and target sequences around the target loci. The gRNA target sequences are indicated by red. The genomic sequences around the target sites of wild type (WT) and Snail CRISPR 1,2 cells. Knockout of Snail and Zeb1 decreases; (**e**) migratory activity and (**f**) drug resistance against taxol (*$p<0.05$ compared with control, Mann-whitney test);

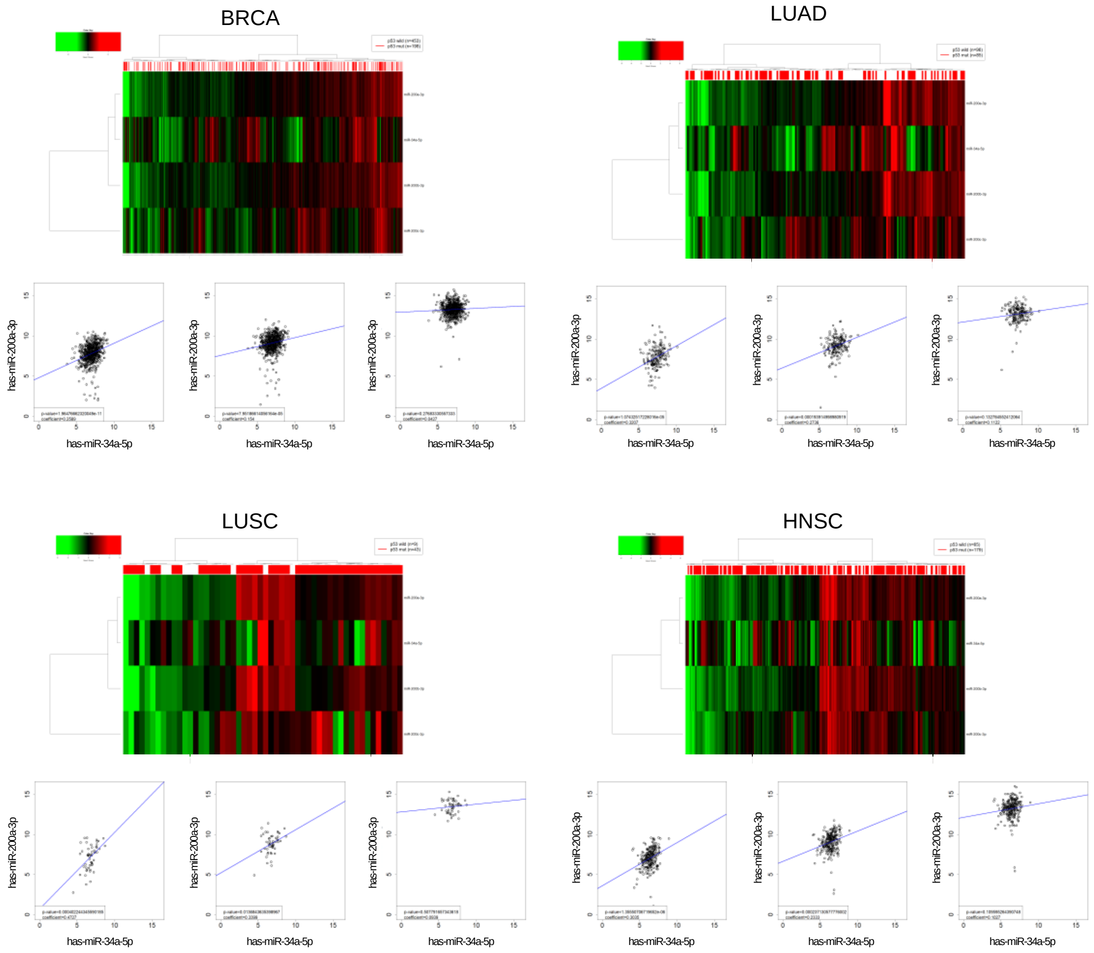

Cancer types	Dataset (n)	Correlation coefficient (*p* value)		
		miR-34a vs miR-200a	miR-34a vs miR-200b	miR-34a vs miR-200c
Breast cancer (BRCA)	n(miRNA ∩ mutation) = 651	0.2589 (*p*=1.96e-11)	0.1540 (*p*=7.95e-05)	0.0427 (*p*=0.2768)
Lung adenocarcinoma (LUAD)	n(mRNA ∩ miRNA ∩ mutation) = 181	0.3207 (*p*=1.07e-05)	0.2736 (*p*=0.0002)	0.1122 (*p*=0.1328)
Lung squamous cell carcinoma (LUSC)	n(mRNA ∩ miRNA ∩ mutation) = 52	0.4727 (*p*=0.0004)	0.3399 (*p*=0.0137)	0.0939 (*p*=0.5077)
Head & neck squamous cell carcinoma (HNSC)	n(mRNA ∩ miRNA ∩ mutation) = 244	0.3035 (*p*=1.35e-06)	0.2333 (*p*=0.0002)	0.1027 (*p*=0.1096)

Figure S2. The correlation of miR-34a and miR-200 family in BRCA, LUAD, LUSC, and HNSC: An supervised hierarchical clustering based on miR-34a and miR-200 family expression profiles in other type of cancers. In the heatmap, red denotes higher relative expression, whereas green indicates lower relative expression, with degree of color saturation reflecting the magnitude of the log expression signal.

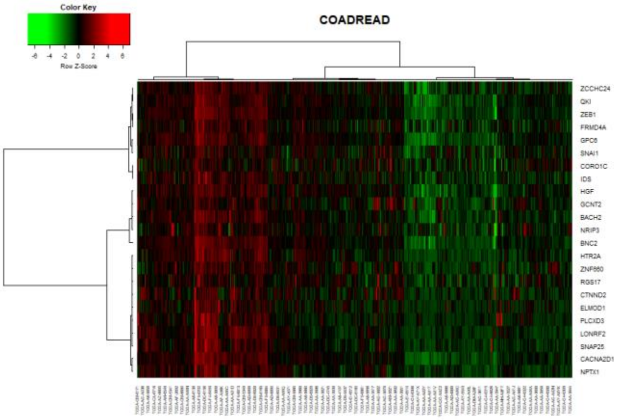

Figure S3. Co-target genes of Snail and Zeb1 in COADREAD samples:
An supervised hierarchical clustering of mRNAs associated with Snail and Zeb1 expression profiles

Figure S4. Uncropped images of all the western blot data

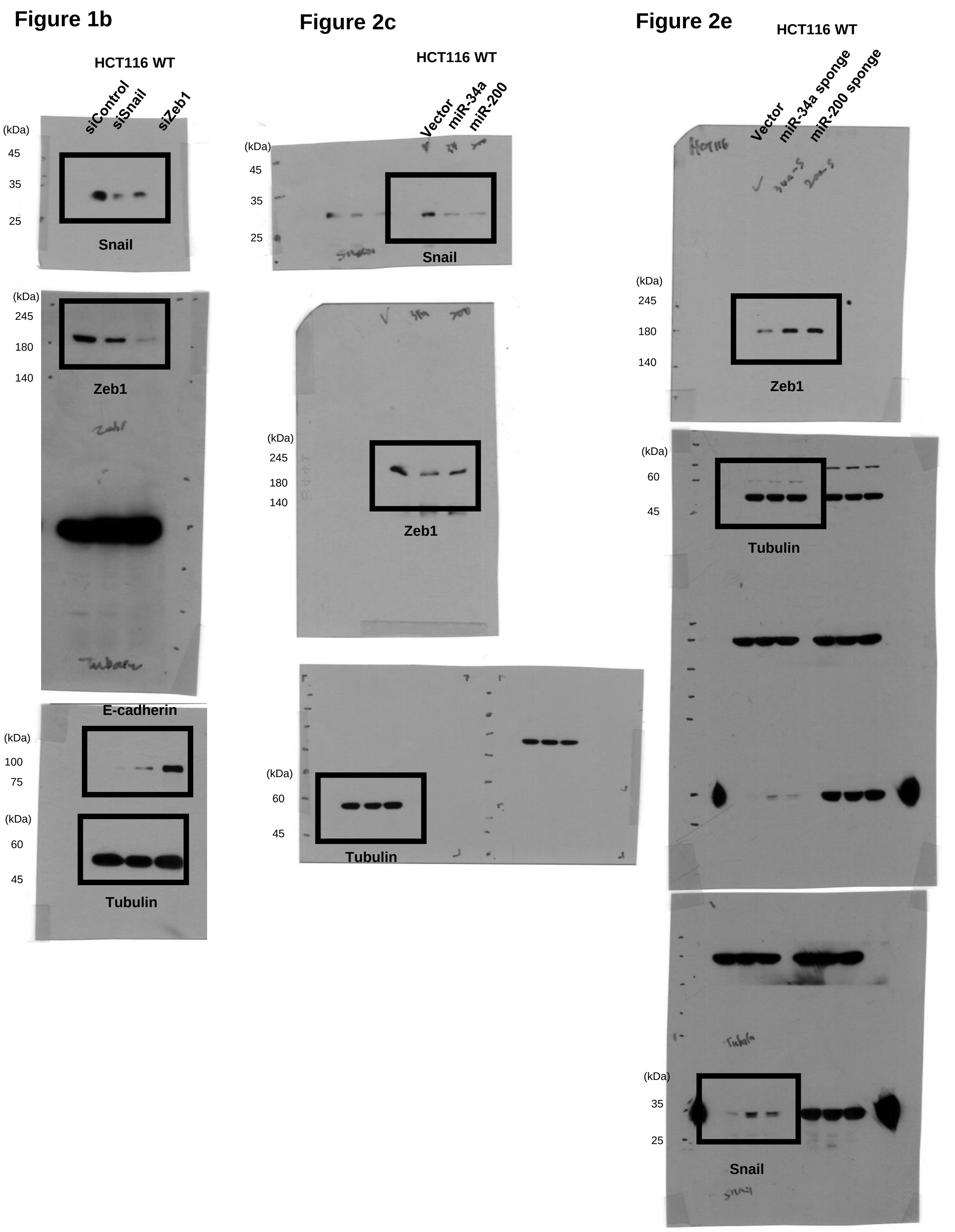

Figure S4. Uncropped images of all the western blot data

Figure 3D

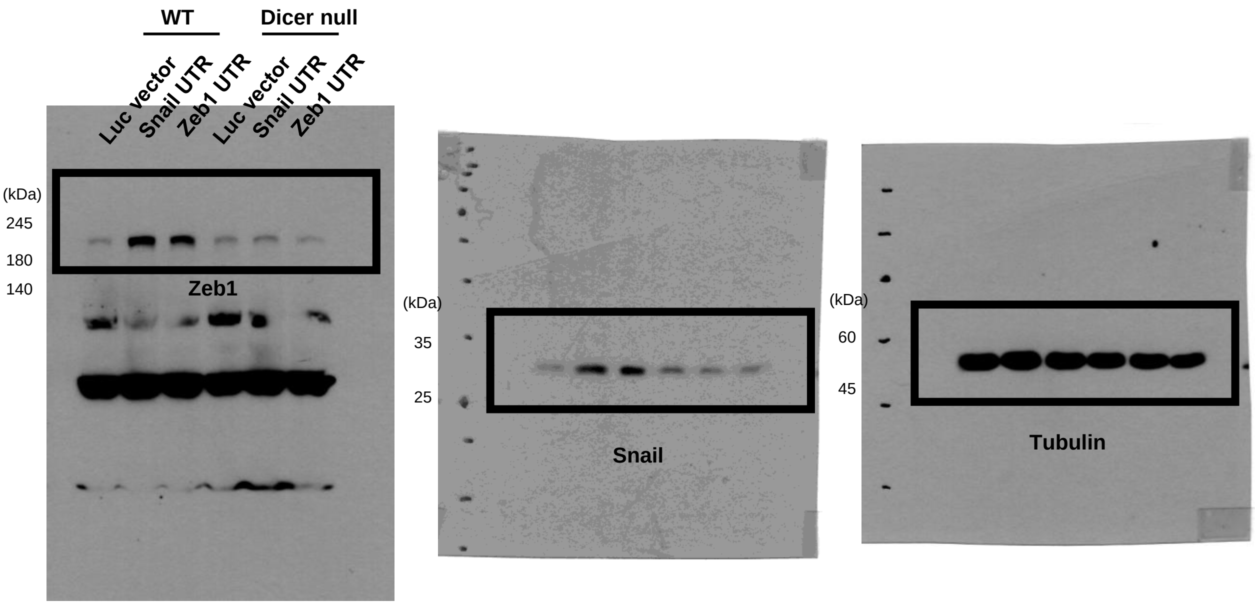

Figure S1C

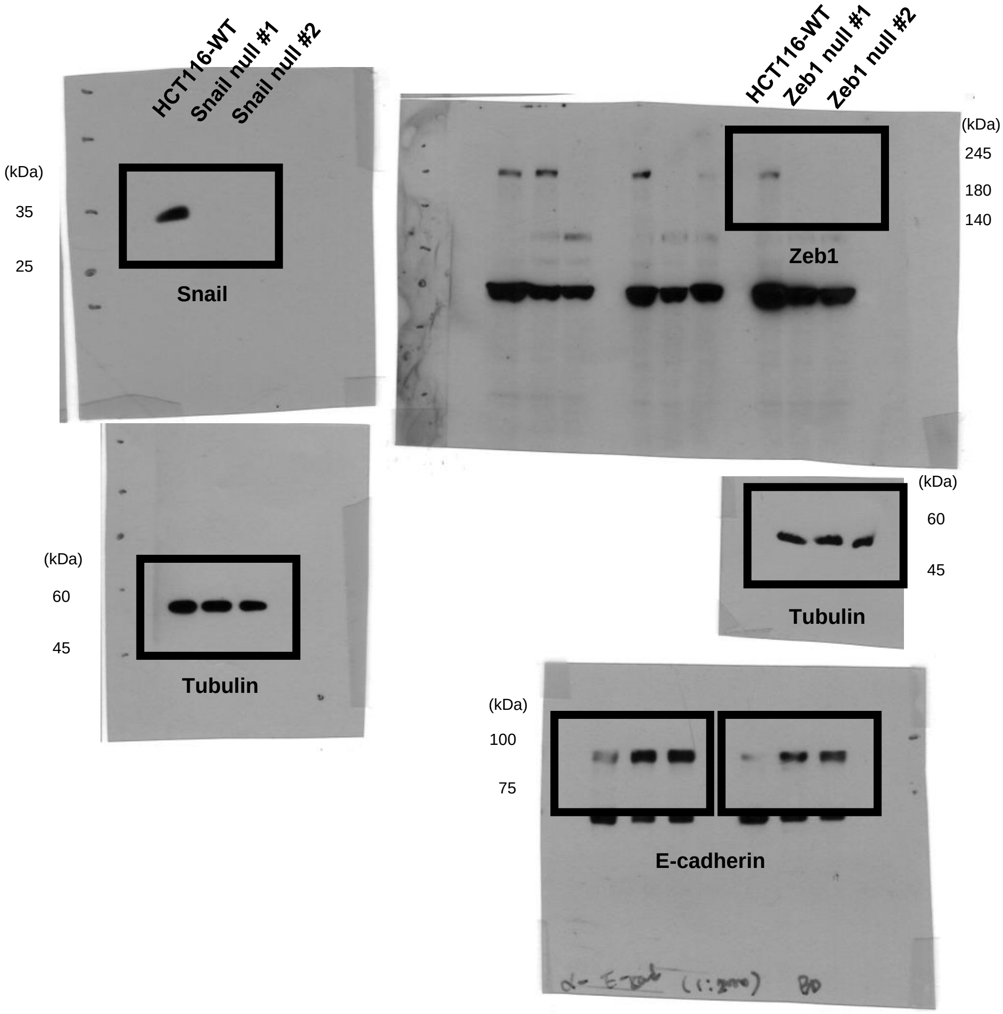

Table S1. Correlation score between miRs and mRNAs

Gene	miR-34a-5p	miR-200bc-3p	miR-200a-3p	score.sum
ZEB1	-0.02	-0.86	-0.76	-1.64
ELMOD1	-0.69	-0.1	-0.45	-1.24
PRKACB	-0.28	-0.5	-0.42	-1.2
YWHAG	-0.16	-0.51	-0.53	-1.2
CCNE2	-0.59	-0.23	-0.34	-1.16
PPP4R2	-0.36	-0.59	-0.18	-1.13
CALCR	-0.58	-0.27	-0.21	-1.06
E2F3	-0.47	-0.25	-0.34	-1.06
RALGPS2	-0.5	-0.1	-0.44	-1.04
BNC2	-0.43	-0.4	-0.19	-1.02
CTNND2	-0.42	-0.15	-0.4	-0.97
HSPA13	-0.27	-0.33	-0.33	-0.93
OLIG3	-0.53	-0.05	-0.3	-0.88
PLAG1	-0.51	-0.12	-0.25	-0.88
CORO1C	-0.46	-0.23	-0.18	-0.87
KLF12	-0.18	-0.23	-0.45	-0.86
ZNF281	-0.56	-0.07	-0.22	-0.85
ZCCHC24	-0.05	-0.29	-0.5	-0.84
NRIP3	-0.6	-0.02	-0.2	-0.82
LASS6	-0.39	-0.29	-0.12	-0.8
C6orf120	-0.08	-0.42	-0.3	-0.8
SGK269	-0.45	-0.15	-0.17	-0.77
ELL2	-0.37	-0.3	-0.08	-0.75
C21orf91	-0.11	-0.31	-0.32	-0.74
DR1	-0.02	-0.18	-0.53	-0.73
CDK6	-0.41	-0.02	-0.27	-0.7
SIRT1	-0.41	-0.08	-0.2	-0.69
HS2ST1	-0.15	-0.21	-0.33	-0.69
SERINC1	-0.25	-0.25	-0.16	-0.66
GAB1	-0.19	-0.11	-0.36	-0.66
DSTYK	-0.27	-0.02	-0.35	-0.64
RGS17	-0.44	-0.01	-0.17	-0.62
FOXP1	-0.44	-0.01	-0.17	-0.62
ITSN1	-0.39	-0.1	-0.13	-0.62
SNAP25	-0.22	-0.4	0	-0.62
RBM33	-0.21	-0.08	-0.33	-0.62
RASA2	-0.31	-0.18	-0.12	-0.61
C4orf31	-0.21	-0.06	-0.33	-0.6
ATXN1	-0.16	-0.17	-0.26	-0.59
GCNT2	-0.37	-0.03	-0.19	-0.59
PLCXD3	-0.29	-0.2	-0.1	-0.59
ALS2CR4	-0.2	-0.24	-0.15	-0.59
GPR12	-0.34	-0.02	-0.2	-0.56
MIB1	-0.14	-0.27	-0.15	-0.56
SFRS13A	0	-0.34	-0.22	-0.56
BCAP29	-0.11	-0.18	-0.26	-0.55
MEGF9	-0.33	-0.01	-0.2	-0.54
MIER3	-0.3	-0.1	-0.14	-0.54
S100PBP	-0.15	-0.22	-0.17	-0.54

Gene	miR-34a-5p	miR-200bc-3p	miR-200a-3p	score.sum
REEP3	-0.2	-0.09	-0.24	-0.53
LYPD6	-0.1	-0.23	-0.2	-0.53
CLOCK	-0.22	-0.06	-0.24	-0.52
PTEN	-0.1	-0.08	-0.34	-0.52
HTR2A	-0.31	-0.05	-0.15	-0.51
KCNJ2	-0.25	-0.03	-0.23	-0.51
ATXN7L1	-0.15	-0.02	-0.34	-0.51
C5orf41	-0.14	-0.01	-0.36	-0.51
TP53INP1	-0.09	-0.12	-0.29	-0.5
ZNF641	-0.33	-0.06	-0.1	-0.49
SLC39A9	-0.21	-0.01	-0.27	-0.49
NFIA	-0.06	-0.28	-0.13	-0.47
HGF	-0.21	-0.01	-0.25	-0.47
DUSP7	-0.08	-0.19	-0.19	-0.46
CALU	-0.06	-0.23	-0.17	-0.46
43897	-0.01	-0.03	-0.42	-0.46
HMGB1	-0.18	-0.11	-0.16	-0.45
WHSC1L1	-0.35	-0.06	-0.04	-0.45
LPGAT1	-0.18	-0.03	-0.24	-0.45
ZNF660	-0.2	-0.1	-0.14	-0.44
PDCD4	-0.25	-0.03	-0.16	-0.44
SFRS12IP1	-0.12	-0.11	-0.21	-0.44
KLF6	0	-0.25	-0.19	-0.44
DCX	-0.36	-0.04	-0.03	-0.43
ARL5A	-0.08	-0.33	-0.02	-0.43
IDS	0	-0.26	-0.16	-0.42
CACNA2D1	-0.21	-0.12	-0.09	-0.42
SLC5A3	-0.27	-0.06	-0.08	-0.41
RNF38	-0.17	-0.11	-0.13	-0.41
BACH2	-0.17	-0.09	-0.15	-0.41
SUGT1	-0.02	-0.2	-0.19	-0.41
NAA50	-0.3	-0.03	-0.08	-0.41
GPC6	-0.22	-0.07	-0.11	-0.4
FAM126B	-0.21	-0.09	-0.1	-0.4
NPTX1	-0.16	-0.18	-0.06	-0.4
HLF	-0.02	-0.22	-0.16	-0.4
CDON	-0.31	-0.02	-0.06	-0.39
FRMD4A	-0.29	-0.05	-0.05	-0.39
FNDC3B	-0.13	-0.06	-0.2	-0.39
MAP3K2	-0.11	-0.14	-0.14	-0.39
CSNK1G3	-0.14	-0.18	-0.06	-0.38
ABL2	-0.08	-0.02	-0.28	-0.38
PANK3	-0.04	-0.02	-0.32	-0.38
QKI	-0.01	-0.18	-0.19	-0.38
RNF11	0	-0.02	-0.34	-0.36
AKAP2	-0.14	-0.06	-0.16	-0.36
LONRF2	-0.13	-0.08	-0.15	-0.36
PGM2L1	-0.12	-0.15	-0.08	-0.35
ASXL3	-0.11	-0.09	-0.15	-0.35
GATSL2	0	-0.35	0	-0.35
